# Development of a *Plasmodium vivax* biobank for functional ex vivo assays

**DOI:** 10.1186/s12936-023-04668-2

**Published:** 2023-08-31

**Authors:** Rashmi Dash, Kristen M. Skillman, Ligia Pereira, Anjali Mascarenhas, Sheena Dass, Jayashri Walke, Anvily Almeida, Mezia Fernandes, Edwin Gomes, John White, Laura Chery-Karschney, Anar Khandeparkar, Pradipsinh K. Rathod, Manoj T. Duraisingh, Usheer Kanjee

**Affiliations:** 1 Goa Medical College and Hospital, Bambolim, Goa 403202 India; 2 Departments of Chemistry and Global Health, University of Washington, Seattle, WA 98195 USA; 3 Department of Immunology and Infectious Diseases, Harvard T. H. Chan School of Public Health, Boston, MA 02115 USA

**Keywords:** *Plasmodium vivax*, Cryopreservation, Short-term culture, Biobank

## Abstract

**Background:**

*Plasmodium vivax* is the second most prevalent cause of malaria yet remains challenging to study due to the lack of a continuous in vitro culture system, highlighting the need to establish a biobank of clinical isolates with multiple freezes per sample for use in functional assays. Different methods for cryopreserving parasite isolates were compared and subsequently the most promising one was validated. Enrichment of early- and late-stage parasites and parasite maturation were quantified to facilitate assay planning.

**Methods:**

In order to compare cryopreservation protocols, nine clinical *P. vivax* isolates were frozen with four glycerolyte-based mixtures. Parasite recovery post thaw, post KCl-Percoll enrichment and in short-term in vitro culture was measured via slide microscopy. Enrichment of late-stage parasites by magnetic activated cell sorting (MACS) was measured. Short and long-term storage of parasites at either − 80 °C or liquid nitrogen were also compared.

**Results:**

Of the four cryopreservation mixtures, one mixture (glycerolyte:serum:RBC at a 2.5:1.5:1 ratio) resulted in improved parasite recovery and statistically significant (P < 0.05) enhancement in parasite survival in short-term in vitro culture. A parasite biobank was subsequently generated using this protocol resulting in a collection of 106 clinical isolates, each with 8 vials. The quality of the biobank was validated by measuring several factors from 47 thaws: the average reduction in parasitaemia post-thaw (25.3%); the average fold enrichment post KCl-Percoll (6.65-fold); and the average percent recovery of parasites (22.0%, measured from 30 isolates). During short-term in vitro culture, robust maturation of ring stage parasites to later stages (> 20% trophozoites, schizonts and gametocytes) was observed in 60.0% of isolates by 48 h. Enrichment of mature parasite stages via MACS showed good reproducibility, with an average of 30.0% post-MACS parasitaemia and an average of 5.30 × 10^5^ parasites/vial. Finally, the effect of storage temperature was tested, and no large impacts from short-term (7 days) or long-term (7–10 years) storage at − 80 °C on parasite recovery, enrichment or viability was observed.

**Conclusions:**

Here, an optimized freezing method for *P. vivax* clinical isolates is demonstrated as a template for the generation and validation of a parasite biobank for use in functional assays.

**Supplementary Information:**

The online version contains supplementary material available at 10.1186/s12936-023-04668-2.

## Background

*Plasmodium vivax* is the most geographically widespread malaria parasite species and is responsible for the second highest burden of malaria globally. While the total incidence of *P.* vivax has decreased globally from 20.5 million cases in 2000 to 4.9 million cases in 2021 [1], significant obstacles remain in efforts to eradicate this pathogen, including the lack of a *P. vivax* vaccine and increasing resistance to anti-malarial drugs.

Research into the fundamental biology of *P. vivax* has been severely limited due to the lack of a continuous in vitro culture system [2–4]. This lack of continuous culture necessitates the use of clinical *P. vivax* isolates for experimental investigations, and limited access to these isolates has hampered progress in advancing knowledge about this parasite. Recently, short-term in vitro culture approaches for *P. vivax* have been established (including in some cases the use of cryopreserved isolates) [3, 5, 6], making it possible to perform single cycle drug resistance assays [3, 7] as well as invasion assays to assess host reticulocyte tropism [8], the effects of invasion inhibitory antibodies [9–16] and host receptor mutants [17, 18]. There is increasing recognition of the importance of biobanking infectious disease samples [19–21], including *Plasmodium falciparum* [22–24]. The generation of a biobank of cryopreserved *P. vivax* isolates that can be reliably thawed for downstream experimentation (functional assays, gene expression assays, genomics) would be a critical resource for further advancements in the field [4].

Cryopreservation of blood stage malaria parasites has a long history with early studies relying on rapid freezing to dry-ice or liquid nitrogen temperatures and rapid thawing of RBCs [25, 26] (reviewed in [27]). These early experiments were performed without cryoprotectants and resulted in low recovery of parasites and high levels of RBC lysis. Most modern methods use glycerol-based cryoprotectant solutions which improved recovery of RBCs [28–31], while a minority have used dimethyl sulfoxide (DMSO) [30, 31] (Additional file 1: Table S1). Several cryopreservation methods for *P. vivax* have been reported [5, 7, 32–36], the majority based on adding various ratios of buffered 57% glycerol/lactate solution (Glycerolyte 57) to packed RBCs. To date however, there has not been a systematic comparison of the effectiveness of these different cryopreservation methods for *P. vivax*, largely due to limited research material from clinical isolates.

Here, a continuous source of patient *P. vivax* isolates from the Goa Medical College and Hospital in Goa, India was utilized to compare the effectiveness of four different cryopreservation approaches. An experimental biobank of cryopreserved *P. vivax* isolates was then established, and these samples were used to validate the efficiency of parasite recovery post thaw, the enrichment of early stage parasites via KCl-Percoll density sedimentation [3, 37], the maturation of parasites in short-term in vitro culture and the enrichment of mature stage parasites post MACS [38]. Furthermore, the effects of temperature cycling between − 80 °C (dry ice/freezer) to − 196 °C (liquid nitrogen) were tested to model the temperature changes that occur during the transportation of frozen samples for short-term and long-term storage. These results will inform future cryopreservation and transportation of *P. vivax* samples and aid in the establishment of cryopreserved biobanks for future experimental endeavors with this understudied parasite.

## Methods

### Ethics approval

The human subject protocol and consent forms for enrolling *Plasmodium*-infected patients in this study at Goa Medical College and Hospital (GMC) were reviewed and approved by the Institutional Review Boards of the Division of Microbiology and Infectious Diseases (DMID) at the U.S. National Institute of Allergy and Infectious Diseases (approval DMID 11-0074), the University of Washington (approval 42271/1192), as well as the Institutional Ethics Committee (IEC) at Goa Medical College Hospital, Bambolim, Goa, India.

### Sample collection

The study was conducted at the Goa Medical College Hospital, at the Malaria Evolution in South Asia-International Centers for Excellence in Malaria Research laboratory. Venous blood was collected in 6 mL vacutainer tubes (acid dextrose solution anticoagulant; BD India, #364606) from all the smear positive patients for *P. vivax* mono infection with signed informed consent between the age of 12 months to 65 years from 2013 to 2019. Pregnant (self-reported) and anaemic patients were excluded from enrollment.

### Sample processing and cryopreservation

Clinical samples that tested positive for *P. vivax* by rapid diagnostic test (FalciVax, Zephyr Biomedicals #50300025, Goa, India) were collected and smears were prepared and stained using Field Stains A (Himedia India, #S008) and B (Himedia India, #S009). Samples with parasitaemia > 0.01% were included for further analysis. The blood was centrifuged at 800*g* for 5 min at room temperature, and serum from each isolate was separated and stored at − 80 °C for related studies. The pelleted blood was washed using phosphate buffered saline (Thermo Fisher Scientific, USA, #J61196.AP) supplemented with 0.5% (w/v) bovine serum albumin (Millipore Sigma USA, #10735086001) to separate any leftover serum components. Prior to cryopreservation, the pelleted blood was separated from leukocytes, by running the sample through CF-11 column filters prepared in the lab. Smears were prepared post processing with CF-11 to assess parasite stages and to examine the leukocyte removal. De-leukocyted samples were cryopreserved using Glycerolyte 57 (Baxter/Fenwal USA, #4A7833), added as follows: glycerolyte 57:serum:RBC ratio was 2:0:1 for mixture 1 [7], 2.5:1.5:1 for mixture 2 [35, 39], 1.66:0:1 for mixture 3 [36] and 4.15:1.5:1 for mixture 4. Samples were stored in cryovials (Fisher Scientific USA, #50001020) at − 80 °C for 24 h prior to long-term storage in liquid nitrogen cryo-tanks at − 196 °C. The serum used in the study was heat-inactivated AB + pooled plasma (Access Biologicals, USA, #A17054) collected from a non-malaria endemic region.

### Short term *P. vivax* culture

*Plasmodium vivax* isolates were thawed using a standard protocol [39]. Samples were thawed out rapidly in 37 °C water bath followed by dropwise addition of 1/10^th^ volume of 12% (w/v) NaCl (Himedia India, #TC046) and 10 volumes of 1.6% (w/v) NaCl. Samples were subsequently pelleted at 500*g* for 5 min and then washed 2 × with IMDM + glutamax media (Thermo Fisher Scientific, USA, #31980030). Parasites were enriched using 1.080 g/mL KCL Percoll gradients [3, 37] (Percoll: Cytiva/Millipore Sigma #17-5445-01; KCl: Himedia India, #TC010). Three milliliters of the sample in media (at between 20 and 25% haematocrit) were layered on an equal volume of 1.080 g/mL KCl Percoll at room temperature in a 15 mL centrifuge tube. The layered sample was centrifuged at 1200*g* for 15 min with slow acceleration and no brake. The enriched parasites from the Percoll interface were washed in IMDM + glutamax + 0.5% (w/v) BSA and were subsequently cultured in IMDM + 10% (v/v) AB + heat-inactivated serum with 0.5%(v/v) gentamycin (Thermo Fisher Scientific, USA #15750060) per condition in 1 mL volumes in 24-well plates (Nunc/Thermo Fisher Scientific, USA, #144530). Haematocrits ranged from 0.2 to 3.5% with an average of 1.8%. The plates were incubated at 37 °C and with mixed gas (5% CO_2_, 10% O_2_ and 85% N_2_ combination) in modular incubator chambers (Billups Rothenberg, USA, #MIC-101) for 24–72 h as needed for the data assessment. Enrichment of parasites via MACS was performed as previously reported [40].

### Microscopy

Slides for the experiments were counted on a light microscope using 100X objective with oil immersion with a 1:4 reticle on a Zeiss Primo Star microscope. The slides were counted and accessed for stages using the whole field method [41] across multiple fields (15–20 fields) until 500 red blood cells were counted in the small box of the 1:4 reticle. The slides for the different time points during the experiment were prepared using a cytospin (Shandon/Thermo Scientific) with 100 µL 10% (w/v) bovine serum albumin in PBS. Parasitaemia was calculated as:

$$\frac{{\# {\text{ infected cells}}}}{{(\# {\text{ RBCs}} \times {\text{ whole field factor}})}} \times 100\%$$

where the whole field factor for the 1:4 reticle was 10.9.

### Data analysis

All the data for the experiments were analysed using Microsoft Excel and Graphpad Prism (version 9.8.14). Comparisons between experimental conditions were performed via one-way ANOVA analysis (or where slide data was missing due to unreadable slides via mixed-effect model analysis). The analysis was performed with matching by parasite isolate, with the assumption of a Gaussian distribution of residuals and assumption of sphericity. Tukey’s multiple comparisons test was used to determine statistically significant differences between individual sets of conditions. Primary data for all results can be found in Additional file 2. 

## Results

### Comparing cryopreservation mixtures

**Fig. 1:**
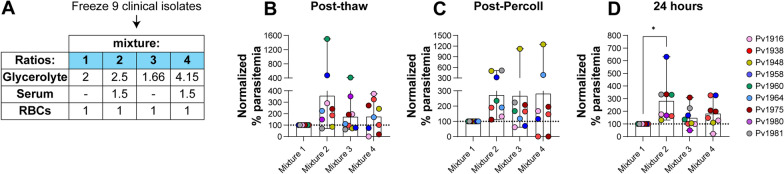
Comparison of four different freezing mixtures. **A** Nine clinical *P.* vivax isolates were cryopreserved using four mixtures with different ratios of RBCs, serum and glycerolyte. Comparison of **B** post-thaw parasitaemia, **C** post KCl-Percoll parasitaemia and **D** 24-h parasitaemia all normalized to mixture 1 values. *P < 0.05 via one-way ANOVA with Dunnett’s multiple comparison test

Among the malaria-positive individuals presenting at Goa Medical College and Hospital (GMC), > 80% are infected with *P. vivax* [42]*.* The ability to collect this number of *P. vivax* isolates has made GMC a unique setting to develop a biobank of cryopreserved parasites for future experimentation. From 2012 to 2016, over 700 *P. vivax* isolates were cryopreserved using the method from Kosaisavee et al*.* [7]*,* where a 2:1 ratio of the cryoprotectant glycerolyte:RBCs was used. Other ratios of glycerolyte:RBCs have been reported in the literature for cryopreservation of both *P. falciparum* and *P. vivax* (Additional file 1: Table S1). Four cryopreservation mixtures, derived from established cryopreservation protocols [7, 34, 36, 39], (Fig. 1A) that varied in the ratios of glycerolyte:RBCs (with or without the addition of serum) were tested in order to identify any differences from the established 2:1 glycerolyte:RBC ratio.

Nine *P. vivax* isolates were split into 4 equal portions, and each was cryopreserved using one of the mixtures. After a minimum of 1 week’s storage in liquid nitrogen, these samples were thawed using standard methods [39] and the parasitaemia determined via slide microscopy post thaw, post enrichment via KCl-Percoll density gradients [3, 37] and at 24 h in short-term ex vivo culture. Parasitaemia values were normalized to the mixture 1 values at each step. Post thaw (Fig. 1B), an increase in relative parasitaemia was observed for mixture 2 but this did not reach statistical significance. Post KCl-Percoll enrichment (Fig. 1C), mixtures 2, 3 and 4 showed higher relative parasitaemia than mixture 1, but again did not reach statistical significance. At 24 h in short-term culture (Fig. 1D), a statistically significant increase in relative parasitaemia was observed for mixture 2 compared to mixture 1 (P = 0.0123, F (1.781, 12.47) = 6.690 via one-way ANOVA with matched data and Dunnett’s multiple comparisons test). Together these data demonstrated that cryopreservation of *P. vivax* isolates with mixture 2 resulted in improved recovery and maturation of parasites and this cryopreservation formulation was chosen for the establishment of an improved biobank.

### Generation and validation of a biobank

**Fig. 2:**
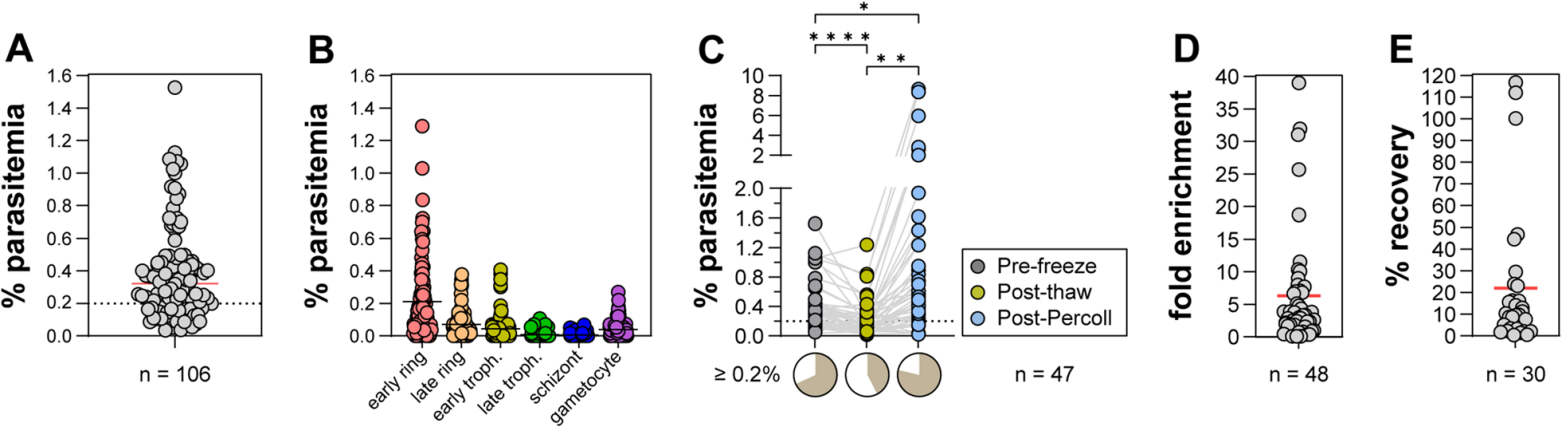
Characterization of biobank and enrichment of rings via KCl-Percoll. **A** Range of pre-freeze parasitaemia values for 106 cryopreserved isolates. **B** Distribution of different stages pre-freeze. **C** Comparison of parasitaemia values pre-freeze, post-thaw and post-KCl-Percoll for 47 isolates. *P < 0.05; **P < 0.01; ***P < 0.001 via One-way ANOVA with Tukey’s multiple comparisons. Lower pie charts show proportion of samples with parasitaemias ≥ 0.2%. **D** Distribution of fold-enrichments post KCl-Percoll. **E** Distribution of the percent recovery of parasites during KCl-Percoll enrichment

Over one hundred *P. vivax* isolates have been cryopreserved using mixture 2, with an average of 8 vials generated per sample (~ 300 µL packed RBCs/vial). The initial parasitaemias ranged from 0.036 to 1.526% with a median of 0.323% and the average of 0.384% (Fig. 2A). Within the collected samples, the mean parasitaemia values were highest for ring stages (early rings (0.212%); late rings (0.071%)) compared to other stages: early trophozoites (0.044%); late trophozoites (0.008%); schizonts (0.005%) and gametocytes (0.040%) (Fig. 2B).

Both drug resistance and invasion assays with *P. vivax* isolates require a sufficient number of parasites for reliable measurements, and as such different strategies have been employed to enrich for parasites either at the ring stage (e.g., for drug resistance assays via KCl-Percoll gradients [3, 7]) or at the schizont stage (e.g., for invasion assays using either Percoll gradients [10] or magnetic enrichment [17, 18, 40]). In a previously reported drug resistance assay with *P. vivax* [3], a minimum of 0.2% parasitaemia was necessary for good quality measurements, and 72% of total isolates met that threshold pre-freeze. In order to characterize any changes in parasitaemia post-thaw and to measure the effect of KCl-Percoll enrichment, the parasitaemia was measured pre-freeze, post-thaw, and post KCl-Percoll enrichment from 47 isolates (Fig. 2C). A statistically significant difference in percent parasitaemia was observed between all three conditions (P = 0.0011 (F (1.034, 47.57) = 11.85) via one-way ANOVA with Tukey’s multiple comparisons). There was an average 74.7% (range 7.21–419.36%) recovery of parasitaemia between pre-freeze versus post thaw and an average of 6.7-fold (range 0.13–39.0-fold) enrichment of parasitaemia post KCl-Percoll (Fig. 2D). Furthermore, the proportion of parasite isolates reaching the 0.2% threshold decreased from 68% pre-freeze to 43% post-thaw but was subsequently increased to 79% post KCl-Percoll (Fig. 2C, lower pie charts) demonstrating the utility of this method for increasing parasitaemia. The percent recovery of parasites during KCl-Percoll enrichment was also estimated by measuring both the percent parasitaemia via slide microscopy and the total RBC numbers via haemocytometer counts both post-thaw and post KCl-Percoll (Fig. 2E). The average recovery was 22% (range 0.43 – 116.8%) suggesting that not all parasites are enriched via this approach.

### Maturation of parasites in short-term ex vivo culture

**Fig. 3:**
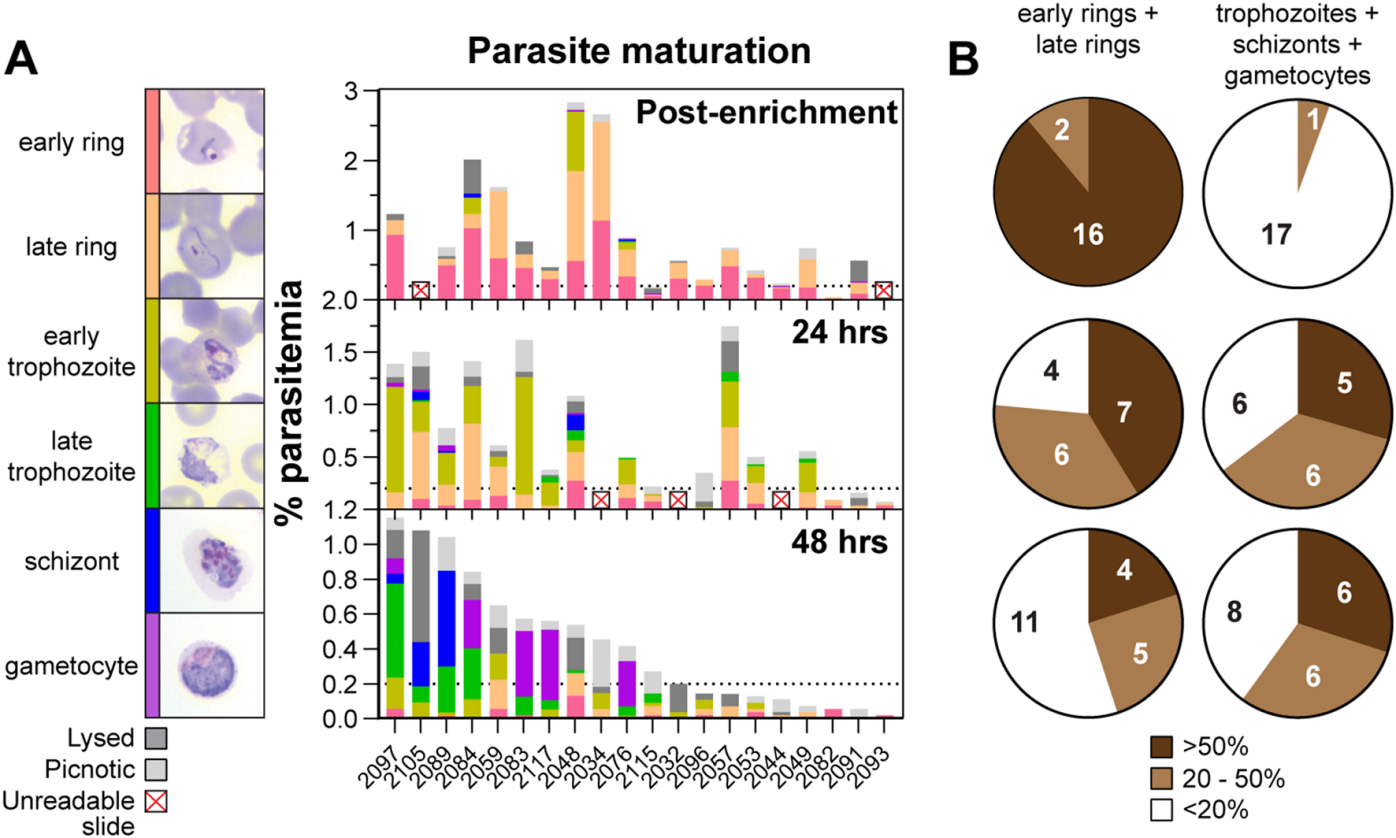
Maturation of parasites in short-term ex vivo culture. **A** Staging and parasitaemia from 20 biobanked samples post-enrichment, 24-h, and 48-h in short-term culture. **B** Pie charts showing proportion of either ring stages (early and late rings) or late-stage (trophozoites, schizonts, gametocytes) parasites across isolates

While there is no long-term culture system for *P. vivax*, short-term ex vivo maturation of parasites has been reported [3, 6, 7, 10, 40]. In order to establish the ability of biobanked isolates to mature, a total of twenty isolates were selected for validation experiments. The samples were thawed and enriched on KCl-Percoll gradients and the staging and parasitaemia were measured post-enrichment and at 24 h and 48 h in short-term culture (Fig. 3A). During the course of these experiments occasional slides were unreadable/damaged, and these isolates were excluded from analysis at the given time point. During the first 24 h, the average parasitaemia remained relatively consistent, with some isolates showing decreases; however, by 48 h there was an average 64.9% decrease in percent parasitaemia across the samples. The maturation of the parasites was quantified by determining the number of isolates with different proportions (< 20%, 20–50%, > 50%) of either immature parasites (early and late rings) or maturing parasites (trophozoites, schizonts and gametocytes) (Fig. 3B). Post enrichment, all isolates had at least 20% immature parasites. At 24 h, 57.9% (11/19) had > 20% mature stages, and a subset of 26.3% (5/19) had > 50% mature stages. These proportions did not change appreciably as by 48 h, 60% of isolates (12/20) had > 20% mature stages with 30% (6/20) having > 50% mature stages.

### Enrichment of mature stages via MACS

**Fig. 4:**
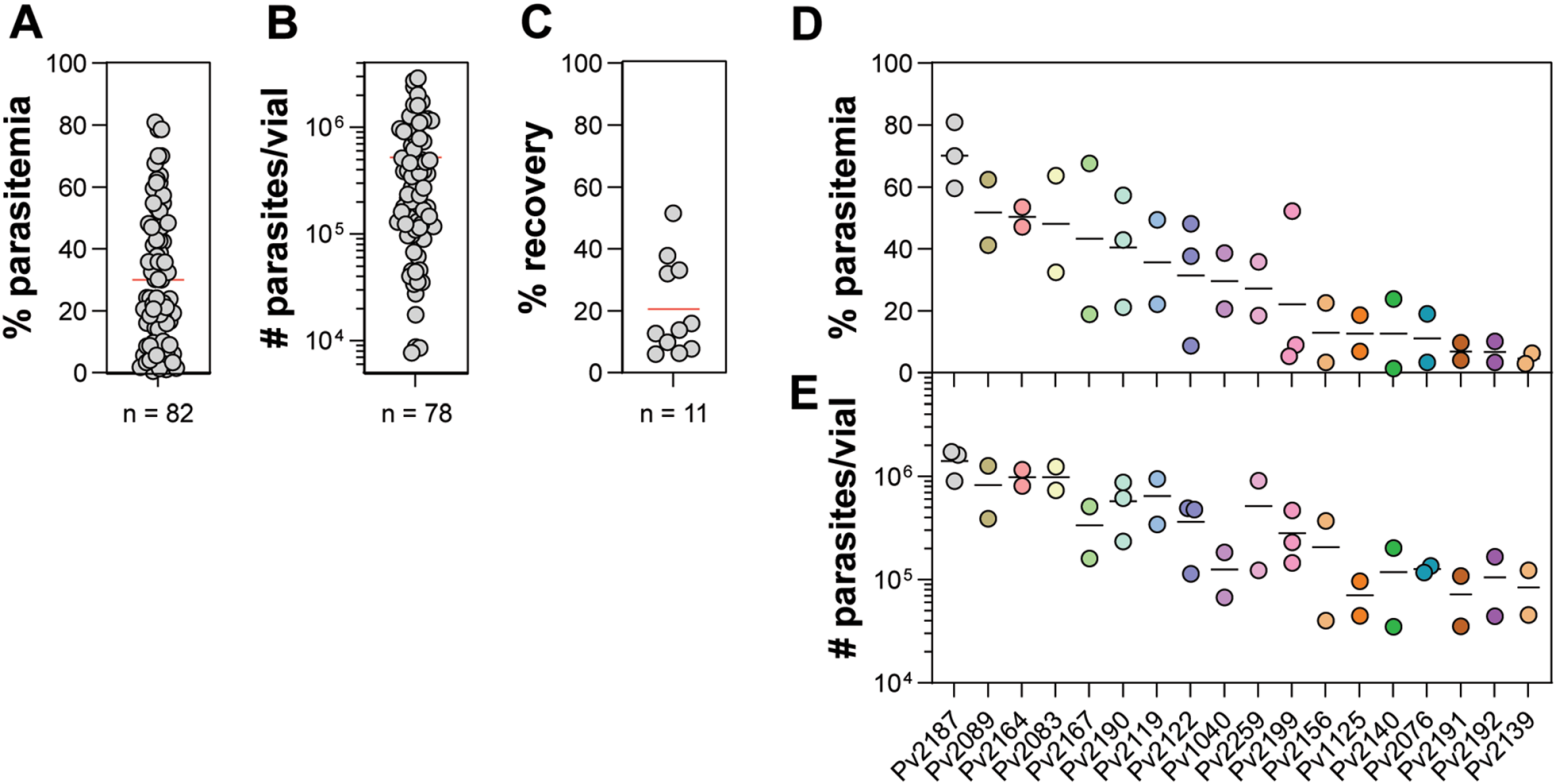
Enrichment of short-term cultured parasites via MACS. Parasite samples were short term cultured for at least 40 h prior to MACS. **A** Distribution of parasitaemias post-MACS enrichment. **B** Calculated number of parasites/vial post-MACS. **C** Calculated % recovery of parasites post-MACS enrichment. Distribution of **D** parasitaemia and **E** number of parasites/vials from repeat thaws of biobanked isolates

Mature parasites can be readily enriched via magnetic activated cell sorting (MACS) through the paramagnetic properties of haemozoin in the parasite food vacuole [38], and is a common method of purifying parasites for use in invasion assays [17, 18, 40]. After at least 40 h of short-term culture, parasites were enriched via MACS (Fig. 4A), with an average of 29.95% parasitaemia (range 0.52–80.81%). In addition, the number of parasites per vial was determined (Fig. 4B), with an average of 5.30 × 10^5^/vial (range 7.68 × 10^4^–2.90 × 10^6^/vial). These values would be useful when using parasites for invasion assays in order to determine the number of assay wells that can be usefully set up per isolate.

The recovery of parasites via MACS enrichment was also estimated by measuring the number of RBCs via haemocytometer and the % parasitaemia via slide microscopy pre-MACS and post-MACS. For the 11 samples that were tested, an average of 20.59% recovery was observed (range 5.94–51.44%) (Fig. 4C). The reproducibility of MACS enrichment and parasite recovery was also determined when multiple independent thaws of the same isolate were made (Fig. 4D, E), and in most cases, the % parasitaemia and the number of parasites per vial varied within less than twofold.

### Impact of temperature variation during transport

**Fig. 5:**
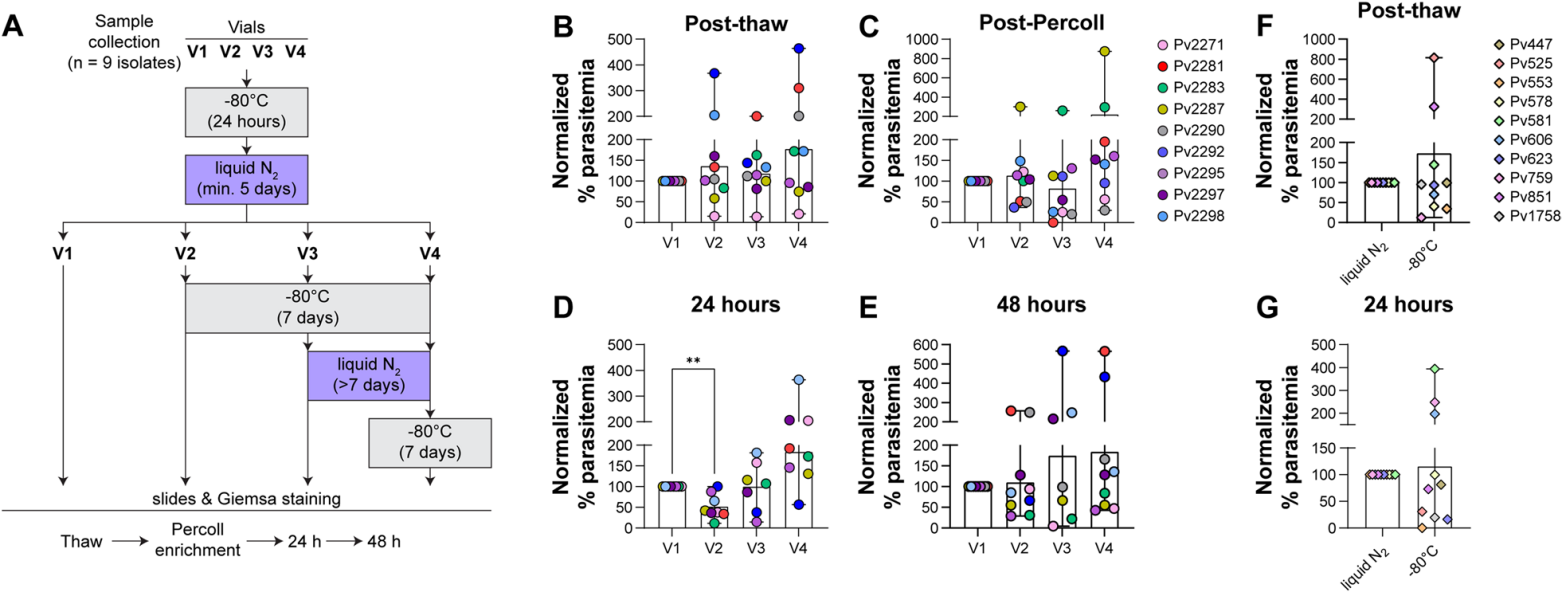
Comparison of freezing and storage conditions. (**A**) Schematic of parasite freezing and storage conditions tested (V1–V4). Comparison of samples Post thaw (**B**), Post-KCl-Percoll (**C**), 24-h (**D**) and 48-h (**E**) with parasitaemia values normalized to V1. **P < 0.01 via mixed effects analysis with Dunnett’s multiple comparisons. Comparison of samples stored long term (7–10 years) in either liquid nitrogen or at − 80 °C: (**F**) post-thaw and (**G**) at 24-h in short-term culture

In addition to the method of cryopreservation, the storage and transport temperature experienced by frozen isolates may impact their viability following thaw. Previous studies with *P. falciparum* have demonstrated reduced viability of cryopreserved parasites stored at − 70 °C compared to liquid nitrogen temperatures [27, 30]. Therefore, a temperature variation experiment was performed to mimic transport of samples from the site of collection to other locations for experimental use. Nine samples were cryopreserved using mixture 2, generating four vials, each of which was subjected to four different temperature storage conditions (Fig. 5A). Vial 1 (V1) was the control condition and was thawed directly from liquid nitrogen within a duration of 5–8 days. Vials V2-V4 (test conditions) were transferred from liquid nitrogen to − 80 °C for 7 days to mimic shipment conditions on dry ice. Vial V2 was thawed after 7 days of storage at − 80 °C while vials V3 and V4 were returned to liquid nitrogen for a further 7 days to mimic possible temperature fluctuation at a site where samples were returned to liquid nitrogen for storage. Vial V4 was cycled a second time to − 80 °C prior to thaw while vial V3 was thawed from liquid nitrogen at 7 days.

Upon thawing, all samples were enriched via KCl-Percoll and short-term cultured with microscopy examination at thaw, enrichment, and following 24 h and 48 h in vitro growth with data normalized to the V1 values (Fig. 5B–E). No statistically significant differences were observed between the conditions post-thaw or post-Percoll. However, a significant reduction in parasitaemia was observed for condition V2 at 24 h (P = 0.010 (F (1.399, 9.330) = 9.024) via multiple effects analysis with Dunnett’s multiple comparisons), although this reduction was not observed at 48 h. Furthermore, in order to test whether there might be long-term effects of storage of parasites at different temperatures, a set of isolates where vials had been stored at both liquid nitrogen and − 80 °C temperatures for between 7 and 10 years were compared (Fig. 5F, G) (note that these samples had been cryopreserved using mixture 1). No statistically significant differences in parasitaemia either post-thaw or at 24 h in short-term culture were observed, suggesting that either storage temperature is capable of preserving parasite.

## Discussion

The establishment of a biobank of cryopreserved *P. vivax* isolates that can be reliably thawed for downstream use would be a powerful tool to facilitate research with this parasite. The current lack of a culture system for *P. vivax* precludes the type of experiments that can be conducted with other culturable *Plasmodium* species, such as *P. falciparum* [43] and *Plasmodium knowlesi* [44–46]. Advances in counting and staging *P. vivax* have been made that would facilitate collection of *P. vivax* for such a resource [3, 41]. Collection of properly preserved and stored isolates, combined with the ability to enrich low parasitaemia samples with the use of KCl-Percoll gradients for ring-stage assays (e.g., drug resistance assays) or to enrich mature parasites via Percoll gradients or MACS (e.g., for invasion assays), would facilitate functional assays with this understudied parasite. Not only would more assays be possible, but the generation of a biobank with multiple freezes from the same donor would allow replicate experiments to be conducted, which is generally not possible when only live isolates can be collected. Finally, establishment of a reliable source of cryopreserved isolates extends the duration of experimentation beyond the typical high-transmission peak season when most clinical isolates are collected.

To inform the generation of a cryopreserved biobank, the progression and maturation of asexual blood stages for *P. vivax* was studied following thaws from vials that had been cryopreserved using four different cryopreservation mixtures with different ratios of Glycerolyte 57 to RBCs (with or without the inclusion of serum). Despite the final concentration of glycerol varying (4.12 M (mixture 1), 3.10 M (mixture 2), 3.86 M (mixtures 3 and 4)) (Additional file 1: Table S1), the four mixtures showed broadly similar characteristics in terms of parasitaemias post-thaw, post KCl-Percoll enrichment and in short-term ex vivo culture. However, mixture 2 was chosen to generate an optimized biobank due to higher recovery and viability of parasites compared to the other mixtures.

Consistent with previous short-term culture optimization experiments [3] a less than 100% recovery of parasites post thaw (Fig. 1C), as well as the presence of picnotic & lysed parasites during in vitro culture was observed (Fig. 3), suggesting that further improvements in the cryopreservation and thawing methodology may be possible, such as comparing the sorbitol method [39] to see if there is improved recovery of parasites compared to the sodium chloride method currently in use [39]. Furthermore, measurement of red blood cell lysis may also be informative in understanding the effectiveness of different cryopreservation conditions.

The use of density gradients to enrich reticulocytes has been reported previously, with a focus either on Nycodenz [37], aqueous multiphase systems [47] or Percoll [3, 40, 48] approaches. Here the ability of KCl-Percoll density gradients to enrich parasites post thaw to parasitaemias sufficiently high for drug resistance assays (> 0.2% parasitaemia [3]) was demonstrated (Fig. 1C). The measured recovery of parasites using this method is still relatively low (Fig. 1E), suggesting that other improvements to this approach may be possible. Future experiments should also directly measure the recovery of reticulocytes (both uninfected and infected) as well as the effectiveness of recovery and maturation of sexual stages [49], which may be useful for future transmission studies.

Measurement of the viability and maturation of parasites during short-term in vitro culture (Fig. 3) suggests that at least 60% of isolates in the biobank will be capable of complete maturation (with at least 20% mature stages). This may be an underestimate as schizonts in some isolates may have already matured and egressed by the 48-h timepoint when slides were made. A more accurate assessment of maturation may require the use of egress inhibitors to block parasite egress [50]. In addition, future comparisons of short-term culture efficiency with live isolates may also be informative. The MACS enrichment of mature parasites (trophozoites, schizonts and gametocytes) showed a relatively wide distribution in parasitaemias, number of parasites/vial and % recovery post MACS. The less than 100% recovery may be due to the wide distribution of parasite stages observed during short-term ex vivo culture (Fig. 3). In addition, the enrichment of parasites between independent thaws showed relatively good reproducibility (Fig. 4D, E) allowing for some ability to predict the number of mature parasites expected from an individual thaw which would be key in planning invasion assays [17, 40, 51].

While long-term storage at liquid nitrogen is preferred, little is known about the effect of cycling between liquid nitrogen and dry-ice/− 80 °C storage temperatures on the viability of *Plasmodium* spp. parasites [27]. When parasite isolates are collected from endemic settings specifically, the available options for storage of samples during transport from the collection point to long-term storage may be limited. The short-term storage of isolates at different temperature regimes showed no significant difference in parasite viability post-thaw and post-enrichment, while a reduction in parasitaemia at 24 h in short-term culture for parasites that had been stored at − 80 °C was noted (Fig. 5D). However, a comparison of long-term storage of samples in liquid nitrogen and at − 80 °C did not reveal any statistically significant differences between storage at either temperature, although it is possible that viability may vary from sample to sample.

## Conclusions

Four different cryopreservation methods for *P. vivax,* and were compared, and one method to provided improved parasite recovery and maturation in short-term in vitro cultures. Using this method, a biobank of clinical isolates was established with multiple freezes per isolate and validated by showing the ability to enrich parasites post-thaw with KCl-Percoll gradients, and for at least 60% of parasites to successfully mature in short-term in vitro cultures. Finally, the tested the storage of cryopreserved parasites at different temperatures (liquid nitrogen storage and − 80 °C storage) was tested, but at least for the duration of this study, no evidence of changes in the viability of parasites stored in liquid nitrogen versus − 80 °C was found.

### Supplementary Information

**Additional file 1**. Summary of published *Plasmodium* spp. cryopreservation methods.

**Additional file 2**. Data for figures 1–5.

## Data Availability

The datasets used and/or analysed during the current study are available from the corresponding author on reasonable request.

## Abbreviations

RBC: Red blood cell
MACS: Magnetically activated cell sorting
DMSO: Dimethyl sulfoxide
GMC: Goa Medical College and Hospital
