# Supplementary material for: Development of a Plasmodium vivax biobank for functional ex vivo assays
Source: Malar J. 2023 Aug 31;22:250. doi: 10.1186/s12936-023-04668-2 (PMC10470152; doi:10.1186/s12936-023-04668-2)
Supplement: Supplementary file 1 — Additional file 1. Summary of published Plasmodium spp. cryopreservation methods. [file 12936_2023_4668_MOESM1_ESM.docx]

**Supplementary Table 1:** Summary of *Plasmodium* spp. cryopreservation methods

| **Reference** | **Year** | **Species** | **Freezing Solution** | **Freezing Solution:RBC ratio** | **Final concentration of cryoprotectant** |
| --- | --- | --- | --- | --- | --- |
| [1] | This study | *P. vivax* | Glycerolyte 57^§^ | 1:1 (60% serum; 40% RBC) **(Mixture 2)** | 3.10 M glycerol |
| [2] | This study | *P. vivax* | Glycerolyte 57^§^ | 1.66:1 (60% serum; 40% RBC) **(Mixture 4)** | 3.86 M glycerol |
| [2] | 2017 | *P. vivax* | Glycerolyte 57^§^ | 1.66:1 | 3.86 M glycerol |
| [1] | 2017 | *P. vivax* | Glycerolyte 57^§^ | 1:1 | 3.10 M glycerol |
| [3] | 2015 | *P. falciparum* | Glycerolyte 57^§^ | 2:1 | 4.12 M glycerol |
| [4] | 2013 | *P. knowlesi* | Glycerol/sorbitol^ξ^ | 7:3 | 2.69 M glycerol |
| [5] | 2013 | *P. vivax*; *P. cynomolgi* | Glycerol/mannitol^θ^ | 1:1 | 1.92 M glycerol |
| [6] | 2013 | *Plasmodium* spp. | Glycerolyte 57^§^ | 1.66:1 **(Mixture 3)** | 3.86 M glycerol |
| [7] | 2013 | *Plasmodium* spp. | Glycerol/sorbitol^ξ^ | 1:1 (60% serum, 40% RBC) | 1.92 M glycerol |
| [8] | 2012 | *P. vivax* | Glycerolyte 57^§^ | 2:1 | 4.12 M glycerol |
| [9] | 2012 | *P. vivax* | Glycerolyte 57^§^ | 2:1 | 4.12 M glycerol |
| [10] | 2006 | *P. vivax* | Glycerolyte 57^§^ | 2:1 **(Mixture 1)** | 4.12 M glycerol |
| [11] | 2002 | *P. knowlesi* | Glycerol/mannitol^θ^ | 1:1 | 1.92 M glycerol |
| [12] | 1989 | *P. falciparum* | Glycerolyte^#^ | 4:1 | 4.96 M glycerol |
| [12] | 1989 | *P. falciparum* | 20% (v/v) DMSO | 1:1 | 1.41 M DMSO |
| [13] | 1985 | *P. falciparum*; *P. vivax* | Alsevers solution + 10% (w/v) glycerol | 3:1 | 1.37 M glycerol |
| [14] | 1975 | *P. falciparum* | Glycerolyte^#^ | 1.6:1 | 3.82 M glycerol |
| [15] | 1973 | *P. falciparum*, *P. knowlesi* | 15% (v/v) DMSO, 0.85% (w/v) NaCl | 1:1 | 1.06 M DMSO |
| [16] | 1968 | RBCs | Glycerol/mannitol^θ^ | 1:1 | 1.92 M glycerol |
| [17] | 1957 | *P. falciparum*; *P. ovale* | None | N/A | N/A |
| [18] | 1939 | *P. knowlesi* | None | N/A | N/A |

^§^Composition of Glycerolyte 57: 6.19 M glycerol; 142.8 mM sodium lactate; 4.0 mM KCl, 3.7 mM NaH_2_PO_4_, 8.7 mM Na_2_HPO_4_, pH 6.8

^#^Composition of Glycerolyte: 6.2 M glycerol, 140 mM sodium lactate, 5 mM KCl, NaH_2_PO_4_ to pH 7.4

^θ^Composition of Glycerol/mannitol solution: 28% (w/v) glycerol, 3% (w/v) mannitol, 0.65% (w/v) NaCl

^ξ^Composition of Glycerol/sorbitol solution: 28% (w/v) glycerol, 3% (w/v) sorbitol, 0.65% (w/v) NaCl

^φ^Composition of complete RPMI: RPMI 1640 supplemented with 25 mM HEPES, 0.2% (w/v) NaHCO_3_, 10% (v/v) serum

**REFERENCES:**

1. Mehlotra RK, Blankenship D, Howes RE, Rakotomanga TA, Ramiranirina B, Ramboarina S, Franchard T, Linger MH, Zikursh-Blood M, Ratsimbasoa AC, et al: **Long-term in vitro culture of Plasmodium vivax isolates from Madagascar maintained in Saimiri boliviensis blood.** *Malar J* 2017, **16:**442.

2. de Oliveira TC, Rodrigues PT, Menezes MJ, Goncalves-Lopes RM, Bastos MS, Lima NF, Barbosa S, Gerber AL, Loss de Morais G, Berna L, et al: **Genome-wide diversity and differentiation in New World populations of the human malaria parasite Plasmodium vivax.** *PLoS Negl Trop Dis* 2017, **11:**e0005824.

3. Stanisic DI, Liu XQ, De SL, Batzloff MR, Forbes T, Davis CB, Sekuloski S, Chavchich M, Chung W, Trenholme K, et al: **Development of cultured Plasmodium falciparum blood-stage malaria cell banks for early phase in vivo clinical trial assessment of anti-malaria drugs and vaccines.** *Malar J* 2015, **14:**143.

4. Moon RW, Hall J, Rangkuti F, Ho YS, Almond N, Mitchell GH, Pain A, Holder AA, Blackman MJ: **Adaptation of the genetically tractable malaria pathogen Plasmodium knowlesi to continuous culture in human erythrocytes.** *Proc Natl Acad Sci U S A* 2013, **110:**531-536.

5. Zeeman AM, der Wel AV, Kocken CH: **Ex vivo culture of Plasmodium vivax and Plasmodium cynomolgi and in vitro culture of Plasmodium knowlesi blood stages.** *Methods Mol Biol* 2013, **923:**35-49.

6. Ismail HA: *Freezing of patient isolates and strains with glycerolyte.* Glasgow, UK & Manassas, VA, USA: EVIMalaR & MR4/ATCC; 2013.

7. Moll K: *Freezing and thawing of asexual Plasmodium spp.* Glasgow, UK & Manassas, VA, USA: EVIMalaR & MR4/ATCC; 2013.

8. Noulin F, Borlon C, van den Eede P, Boel L, Verfaillie CM, D'Alessandro U, Erhart A: **Cryopreserved reticulocytes derived from hematopoietic stem cells can be invaded by cryopreserved Plasmodium vivax isolates.** *PLoS One* 2012, **7:**e40798.

9. Borlon C, Russell B, Sriprawat K, Suwanarusk R, Erhart A, Renia L, Nosten F, D'Alessandro U: **Cryopreserved Plasmodium vivax and cord blood reticulocytes can be used for invasion and short term culture.** *Int J Parasitol* 2012, **42:**155-160.

10. Kosaisavee V, Suwanarusk R, Nosten F, Kyle DE, Barrends M, Jones J, Price R, Russell B, Lek-Uthai U: **Plasmodium vivax: isotopic, PicoGreen, and microscopic assays for measuring chloroquine sensitivity in fresh and cryopreserved isolates.** *Exp Parasitol* 2006, **114:**34-39.

11. Kocken CH, Ozwara H, van der Wel A, Beetsma AL, Mwenda JM, Thomas AW: **Plasmodium knowlesi provides a rapid in vitro and in vivo transfection system that enables double-crossover gene knockout studies.** *Infect Immun* 2002, **70:**655-660.

12. Mathai E, Singh B: **A comparative study of two methods for the cryopreservation of Plasmodium falciparum.** *Trans R Soc Trop Med Hyg* 1989, **83:**469-470.

13. Rossan RN: **Cryopreservation of the blood stages of Plasmodium falciparum and Plasmodium vivax for in vivo studies.** *Am J Trop Med Hyg* 1985, **34:**207-208.

14. Diggs C, Joseph K, Flemmings B, Snodgrass R, Hines F: **Protein synthesis in vitro by cryopreserved Plasmodium falciparum.** *Am J Trop Med Hyg* 1975, **24:**760-763.

15. Booden T, Geiman QM: **Plasmodium falciparum and P. knowlesi: low temperature preservation using dimethylsulfoxide.** *Exp Parasitol* 1973, **33:**495-498.

16. Rowe AW, Eyster E, Kellner A: **Liquid nitrogen preservation of red blood cells for transfusion; a low glycerol-rapid freeze procedure.** *Cryobiology* 1968, **5:**119-128.

17. Jeffery GM: **Extended low-temperature preservation of human malaria parasites.** *J Parasitol* 1957, **43:**488.

18. Coggeshall LT: **Preservation of viable malaria parasites in the frozen state.** *Proceedings of the Society of Experimental Biology and Medicine* 1939, **42:**499 - 501.
